# The Effectiveness of Different Additives on Concrete’s Freeze–Thaw Durability: A Review

**DOI:** 10.3390/ma18050978

**Published:** 2025-02-22

**Authors:** Moutaman M. Abbas, Radu Muntean

**Affiliations:** Faculty of Civil Engineering, Transilvania University of Brașov, 500152 Brașov, Romania; engmoutamanabbas@gmail.com

**Keywords:** cement replacement, concrete durability enhancement, freeze–thaw durability, supplementary cementitious materials, sustainable construction

## Abstract

Enhancing concrete’s resilience against freeze–thaw (F-T) cycles is a critical challenge in civil engineering, especially in cold climates, where repeated freezing and thawing lead to structural degradation. This review explores the effectiveness of various additives, including supplementary cementitious materials (SCMs) and chemical admixtures, in improving concrete durability under F-T conditions. Factors influencing F-T resistance include the type and percentage of SCM replacement, the water–cement ratio, pore structure refinement, and air entrainment. The mechanisms by which additives enhance the durability—such as reducing the permeability, improving the microstructure, and increasing the compressive strength—are examined through an extensive review of experimental studies. The findings indicate that manufactured additives, such as silica fume, metakaolin, nano-SiO_2_, and graphene oxide, significantly enhance the F-T durability by densifying the concrete matrix and mitigating internal damage. In contrast, natural additives, including rice husk ash and zeolite, show potential but require optimization to match the performance of industrial SCMs. Additionally, the preparation and treatment methods of these materials play a crucial role in their effectiveness. This review provides insights into optimizing concrete formulations to enhance the longevity and sustainability, offering practical recommendations for the use of SCMs in cold climates.

## 1. Introduction

The use of supplementary cementitious materials (SCMs) to enhance the freeze–thaw (F-T) resistance of concrete has been extensively researched. Determining the service life of concrete in cold regions depends significantly on the freeze–thaw durability of concrete. This factor holds significant importance in assessing the longevity of concrete in such areas [1]. These additive SCMs, used in the cement industry, are typically composed of natural pozzolans, industrial waste products, and activated minerals. When these materials are used alone or in contact with water, they do not exhibit significant hydraulic reactions with cementitious properties. However, they do possess either pozzolanic or hydraulic characteristics [2].

SCMs have been commonly used in concrete manufacturing. An extensive amount of data has been obtained regarding their compositional properties and how they affect cement hydration and concrete properties. These industrial byproducts from steel production and coal combustion, for example, have unique properties that enhance the workability, durability, and strength within concrete mixes [3]. The extensive use and research of these materials has enabled a deep knowledge on their effects over the aspects of concrete performance; thus, optimized concrete formulations can be performed.

According to the United Nations (UN) Department of Economic and Social Affairs, it is expected that a majority, that is, 70%, of the global population will live in urban areas by the year 2050 [4]. Therefore, it becomes very important to focus on the sustainability of concrete infrastructure in the current scenario. The enhancement of the life of concrete infrastructure is necessary for improving sustainability. In cold climates, frost damage is one of the important factors that affects the durability of concrete [5].

Freeze–thaw damage represents one of the main mechanisms of the destruction of concrete structures in environments with alternately repeating freezing and thawing. It originates from pore water expansion within concrete during freezing, leading to crack and spall formation. Additives are another type of admixture which improves the freeze–thaw resistance of concrete. The additives have different functions, acting by reducing the permeability of the concrete, increasing its strength, and enhancing its ability to resist crack propagation [6].

**Figure 1:**
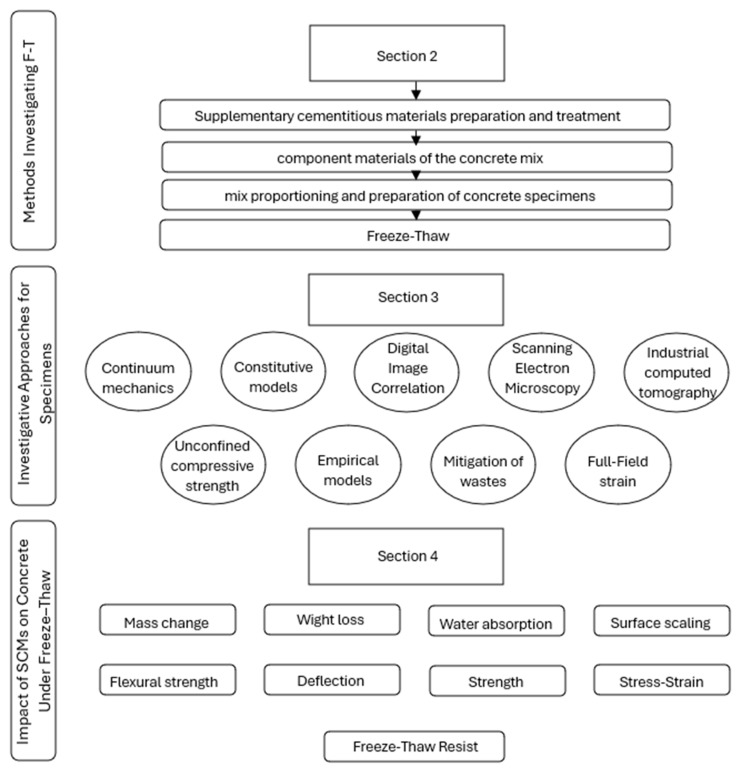
Flow chart reviewing the effectiveness of different additives on concrete’s freeze–thaw durability.

The present general review paper will look at the current information available on the effect of a wide range of SCMs on concrete resistance to freezing and thawing; the structure of the paper is shown in Figure 1. Also described in this paper are the mechanisms by which these different types of additives work, and the results of several research studies that have tested them. This paper will further discuss the factors that affect the performance of additives and some of the recommendations that have been provided for the use of additives in concrete mixes. Despite that, SCMs have an environmental effect, because the availability of industrial byproducts is slowly declining, for example, in the case of fly ash. This has, therefore, spurred investigations for more sustainable alternatives, whereby natural biomaterials and their byproducts look promising for reducing the environmental footprint of concrete. Such materials include rice husk ash, coconut shell powder, and bamboo ash as possible SCM replacements because of the pozzolanic properties they contain and their abundance as an agricultural waste. These natural materials will not only help to reduce waste but will also be capable of improving the concrete strength and durability.

A major existing gap in the knowledge is the specific impact of these natural SCMs on the freeze–thaw durability of concrete. The established improvement from concrete use with traditional SCMs has been through the improved resistance to freeze–thaw cycles. How natural biomaterials in concrete affect the performance of concrete under such conditions is largely unknown. The studies of how these materials influence the freeze–thaw performance of concrete in regions that are most susceptible to harsh weather are warranted. This paper therefore reviews the potential of natural biomaterials and their byproducts as supplementary cementing materials, with an emphasis on their potential to improve the resistance to freeze–thaw cycles in concrete. Such interfaces of sustainability with performance would go a long way in actualizing more eco-friendly and sustainable concrete solutions.

## 2. Methods Investigating F-T

**Figure 2:**
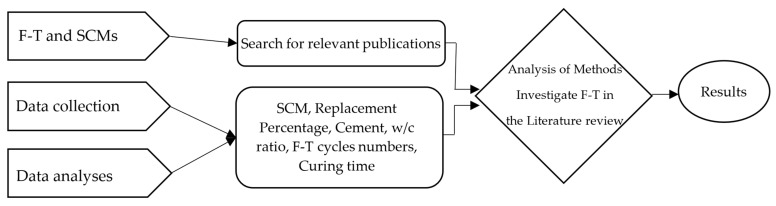
The paper’s methodology (influenced by [7,8]).

This review was conducted using a literature search focusing on the F-T durability of concrete incorporating different additives. The research strategy involved querying major scientific databases, to identify the most relevant peer-reviewed articles. In Figure 2, the method used to ensure comprehensive coverage is shown:

Concrete critical factors influence its overall performance and durability. Standardized test methods (see Table 1), such as ASTM C457 [9], for determining the pore spacing factor provide a more quantifiable measure of the pore structure; the pore structure in hardened concrete is the interconnected network of voids within the material.

1. Porosity is the ratio of the volume of voids in a material to the total volume of the material. It is a measure of how much empty space there is in a material [10,11,12].
2. Aeration is the process of introducing air into fresh concrete. This can be performed by using a variety of methods, such as adding air-entraining admixtures or using a mechanical mixer [10,11,12].
3. The pore spacing factor is a measure of the average distance between pores in a material. It is determined by a standardized test method, such as ASTM C457 [9,10,11,12].
4. Dmax is the maximum size of aggregate particles in concrete [10,11,12].

In his study, Shinkafi, A. B. [13] focused on the meticulous preparation of pozzolanic materials, especially flash-calcined lateritic clay. The calcination conditions, including the temperature and time, were key to the reactivity and characteristics of the clay, thereby further enhancing the pozzolanic potential. The mix included high-strength Portland cement, aluminosilicate sources, alkali metal sources, and aggregates, all carefully selected for their properties. Different mixes, including geopolymer mortars and concretes, were proportioned based on specific ratios, thus ensuring reproducibility. The study found significant pozzolanic activity (SAI > 0.75), and that geopolymer concrete exhibited better freeze–thaw resistance than Portland cement concrete, with less than 5% mass loss after 300 cycles.

In the study by Zhang, S. et al. [14], the preparation of pozzolanic materials, particularly ultrafine metakaolin, was crucial. Metakaolin, obtained from calcined kaolin clay, was finely ground to achieve a high specific surface area (>20,000 m^2^/kg) and high pozzolanic activity (>99% SiO_2_ and Al_2_O_3_). This metakaolin, along with silica fume, was added to a concrete mix that included Portland cement according to the International Organization for Standardization (ISO), as well as sand, river sand, crushed limestone, and a polycarboxylate superplasticizer (SP), for optimized workability. Then, they tested the various concrete mixes with replacements of 9% and 15% metakaolin, comparing them to mixes with silica fume. The results showed that metakaolin significantly reduced the concrete porosity, improving the resistance to chloride ion penetration and freeze–thaw cycles. Silica fume had similar effects, highlighting the durability benefits of both admixtures.

Cheolwoo et al. [15] investigated the freeze–thaw resistance of concrete containing acid-leached rice husk ash (RHA). They suggested that, since RHAs are essentially microstructures with large empty spaces, the air void spacing would be reduced. They prepared concrete mixes with varying percentages of RHAs and silica fume, and then exposed them to freeze–thaw cycles to test the compressive strength and durability. The concrete mix consisted of American Society for Testing and Materials (ASTM) Type I Portland cement, silica fume, acid-leached RHA, polycarboxylate superplasticizer, crushed granite, and river sand. The mixes were prepared with different water-to-binder ratios of 0.38 and 0.45, and then were exposed to 300 and 600 freeze–thaw cycles. At the beginning, the freeze–thaw resistance of the RHA concrete was slightly lower than that of the silica fume concrete, while it was comparative after 600 cycles. The RHA concrete showed excellent compressive strength, without any significant degradation noted up to 56 days and 300 cycles. Thus, the authors concluded that RHA is a potential pozzolanic material in improving the freeze–thaw resistance of concrete; further study may help to deliver other benefits, like higher strength and reduced impacts on the environment.

Nagrockiene and Girskas [16] investigated the influence of a synthetic zeolite admixture on concrete properties. In this work, synthetic zeolite produced using sodium hydroxide and alumina hydroxide at 95 °C was added to concrete made with I 42.5R cement (CEM). The research focused on changes in the density, water absorption, compressive strength, porosity, freeze–thaw resistance, and microstructure. The tests, including compressive strength measurement, scanning electron microscopy (SEM), X-ray diffraction, and porosity analysis, proved the increase in the properties of the concrete by synthetic zeolite. It reduced the water absorption, increased the compressive strength, improved the freeze–thaw resistance, and decreased the porosity. An improved microstructure characterized the benefits accruable from this treatment, which resulted in a denser concrete with the formation of new crystalline phases, such as C3AH6 and C4AH12. The addition of synthetic zeolite, therefore, greatly improved the durability and performance of the concrete.

Ming-hui and Yuan-feng [17] used ordinary silicate cement as the primary binder, with crushed stone (5–25 mm) as coarse aggregates, natural sand as fine aggregates, and fly ash as a supplementary cementitious material. Twenty-four (300 × 100 × 100 mm) prisms were cast with mix proportions. The specimens were cured for 28 days and then immersed in water for 60 days. A freeze–thaw cycling test, following the Chinese standard for test methods of long-term performance and durability, GBJ82-85 (NSPRC 1997), subjected the specimens to temperatures ranging from 6 °C to −15 °C for up to 125 cycles. The test results showed a significant degradation in the compressive strength, elastic modulus, and relative dynamic elastic modulus, particularly after 30 cycles, with varying failure modes observed.

The study by Wawrzenczyk et al. [18] assessed the freeze–thaw resistance of concrete modified by varying the water-to-binder (W/B) ratio, Ground Granulated Blast Furnace Slag (GGBS) content, and air entrainment using polymer microspheres. Two series of concrete, both air-entrained and non-air-entrained concretes, were prepared. The tests revealed that higher W/B ratios increased the water absorption and permeability, thereby reducing the compressive strength and freeze–thaw resistance, particularly in the non-air-entrained concrete. The addition of GGBS, especially at a GGBS/C ratio of 0.5, significantly reduced the permeability and improved the freeze–thaw resistance. Air entrainment improved the frost resistance but slightly reduced the compressive strength.

The study by Raed M. Abendeh et al. [19] investigated concrete mixes incorporating waste soda–lime glass as a partial replacement for Glass Aggregate (GA) and Glass Powder (GP). The glass was finely crushed, sieved, and characterized as amorphous via X-ray diffraction. The concrete mixes varied in the GA and GP percentages, with a constant water/cement ratio of 0.5 and the inclusion of a superplasticizer for workability. The specimens were cast and cured for 7, 28, and 60 days. Freeze–thaw tests, conducted per the Standard Test Method for Resistance of Concrete to Rapid Freezing and Thawing (ASTM C666), showed that the inclusion of GP or GA reduced the mass loss compared to the reference specimens, with GP being more effective. The study concluded that waste glass improved the compressive strength and frost resistance, with GP having a more significant positive impact.

Yutao Bi et al. [20] conducted a study on the mechanical properties of ultra-high-molecular-weight polyethylene (UHMWPE) fiber-reinforced Engineered Cementitious Composites (ECCs) designed for link slabs for Northeast and South China. The mixture was composed of P-II 42.5R Portland cement, fly ash, quartz sand, polycarboxylate superplasticizer (PS), and UHMWPE fibers at 2% of the Engineered Cementitious Volume of the composite ECCs. Mechanical tests, including compression and tensile tests, and tests conducted on the UHMWPE-ECC specimens, including bending tests, were performed. The freeze–thaw simulated cycles resulted in 15 years of service in Northeast China, inducing slight surface damage and mass loss, and loss in the compressive strength, tensile strain, and bending strength. In the case of carbonation, the compressive strength in the southern region was enhanced. The strength, tensile strain, and bending strength of the same period were determined, and a SEM analysis was conducted. It was concluded that carbonation actually enhanced the interfacial bond between the UHMWPE fibers and the cement matrix by roughening the fiber surface.

The study by Shuldyakov et al. [21] focused on the preparation and treatment in high-weight concrete with medium alumina cement, granodiorite gravel, coarse sand silica fume, and the following two superplasticizers: Glenium ACE 430 was taken as a polycarboxylate superplasticizer, and SP-1—as a naphthalene-formaldehyde superplasticizer. The water–cement ratio for all of the mixes was maintained at a constant; for one mix, the cement content was high (up to 400 kg/m^3^) so to achieve a maximum strength, while, for the other, the fluidity was maintained. Two sets of concrete specimens were prepared, differing only in the type of superplasticizer used. Standardized freeze–thaw tests were carried out; from the results, it appeared that Glenium ACE 430 improved the freeze–thaw resistance and raised the concrete grade from F_2300_ to F_2400_. By contrast, the SP-1-modified concrete exhibited a dramatic loss in strength after just 37 freeze–thaw cycles, while, at 55 freeze–thaw cycles, almost 50% of the compressive strength was lost. Water absorption tests indicated that the SP-1-modified concrete was more porous after freezing and thawing, while the Glenium ACE 430-modified concrete remained stable.

Kyong-Ku Yun et al. [22] assessed the impact of the latex content and water–cement ratio (w/c) on the durability of very-early strength latex-modified concrete (VESLMC). The investigation included varying latex contents from 0% to 20%, three *w*/*c* ratios (0.36, 0.38, and 0.40), and a fixed cement content of 390 kg/m^3^. The materials used were very-early strength cement, a styrene–butadiene latex polymer emulsion, crushed limestone, and natural sand. The concrete mixing and curing procedures followed strict protocols to ensure consistency. Performance tests showed that increasing the latex content generally improved the durability, including reduced water absorption, enhanced freeze–thaw resistance, and increased flexural strength, though the compressive strength slightly decreased. The study also found that higher latex concentrations enhanced the freeze–thaw resistance, even with a reduced air content, and improved the de-ice salt scaling resistance. These findings highlight the effectiveness of latex modification in improving the durability of concrete in harsh environmental conditions.

The manuscript by Fang Liu et al. [23] detailed the preparation and treatment of materials for an experimental study on concrete, focusing on nanoparticles (SiO_2_ and TiO_2_). Then, the freezing and thawing resistance of the concrete was evaluated by computing the mass loss ratio by industrial computed tomography (CT). The authors also described the properties and mixing methods for incorporating the nanoparticles. The mass loss rate of the specimens was measured after being exposed to specific freezing and thawing cycles. As the number of these cycles increased, the mass loss rate gradually rose, reflecting the progressive deterioration of the cement paste. However, the rate at which the mass loss increased slowed down as the number of freezing and thawing cycles continued to grow. Comparing the 30/15 nm ratios used in the study, the 30 nm of nano-SiO_2_ and 15 nm of nano-SiO_2_ had a better effect in improving the internal structure of the concrete, and the 30 nm of nano-TiO_2_ had a better effect in preventing pore and crack expansion. The results highlight the role of nanoparticles in enhancing the frost resistance and durability of concrete under freeze–thaw conditions.

The research by Ding et al. [24] used clayey soil stabilized with cement and polypropylene (PP) fibers, documenting the effects of freeze–thaw cycles. The materials that were used included clay soil from the Daqing region, China, 32.5R cement as a stabilizing agent, and PP fibers for reinforcement. Tests on single-phase clayey soil were made to characterize the specific gravity, Atterberg limits, maximum dry density, and particle size distribution. PP fibers that were hydrophobic and non-corrosive in nature were added in various proportions to achieve strength and stability. The mix was prepared with three types of cured samples with different cement and fiber percentages. The soil was set at its optimal moisture content through the addition of water and then provided with the cement followed by the PP fibers. At the maximum dry density, the specimens exhibited 1.92 g/cm^3^, having been compacted and then cured for seven days before freeze–thaw cycling was started, which was conducted under controlled temperature conditions. The objective of the research presented was to understand the changes in the mechanical properties of stabilized soils due to freeze–thaw cycles. It was observed that an increased cement content and the number of freeze–thaw cycles contracted the cement-stabilized soil and expanded the fiber-stabilized soil with high contents of randomly distributed fibers. The unconfined compressive strength (UCS) showed a significant decrease after freeze–thaw cycles, which has quite an importance in relation to the use of these effects in soil stabilization—especially in cold regions. The overall results contribute to the production of more durable infrastructural materials for both cold and warm environments in freeze–thaw conditions.

The study by Samer, G. et al. [25] considered some novel materials, for instance, hemp and recycled aggregate concrete (HRAC). The hemp fibers were treated with alkali, silane, and acetyl, and applied to enhance their interaction with the surrounding matrix of concrete. Recycled concrete aggregate (RCA) had to be used, and it had to be appropriately treated to overcome the problems relating to the porous nature of these materials, as well as the generally inefficient bond capabilities, before useful treatment was performed, wherein the aggregates were carefully cleaned and the porous products, like mortar and fine particles, adhering to these materials were screened off. The research work explored numerous combinations with variations in the parameters, such as the percentage of RCA, the Maximum Size Aggregate (MSA), the length of the hemp fiber, and surface treatments, to analyze the impact on concrete properties. The freeze–thaw resistance of HRAC turned out to be similar to that of normal concrete after 144 cycles, and it was shown that these mixes could be a potential durable and sustainable option for use in cold climates.

In the work by Yan-Ru, Z. et al. [26], basalt fibers were used to reinforce concrete samples in order to increase their resistance, not only to plain bending, but also for numerous specific conditions, including cycles of freezing and thawing. These fibers were mixed with the usual ingredients of concrete, such as sand, stone, and cement, with the help of a specifically defined procedure that guaranteed its uniform integration and the provision of stable properties. The concrete specimens were also cured for 120 days to attain ultimate material qualities. Besides basalt fibers, other standard materials, like sand, stone chips, cement, and water, were also used in their usual proportions so to gain the desired characteristics. The standard grade used for concrete purposes, C30, was also mixed with differing densities of basalt fibers to check their effect on the performance. Further, the specimens were subjected to freeze–thaw cycles at frequencies of 0, 15, 30, and 60 times to simulate real-world conditions. It was observed that freeze–thaw cycles substantially decreased the elastic deformation capability and the impact resistance ability of the concrete, which suggests that environmental factors need to be considered in the design of concrete classes.

In the study by A. Mohammed et al. [27], high-quality cement and graphene oxide (GO) were used to enhance the concrete performance. GO was first dispersed in water to form a stable suspension, ensuring its even distribution in the cement matrix, which is crucial for improving the freeze–thaw resistance and compressive strength. The mix included standard Portland cement, fine aggregates, and water, with GO added at varying amounts, up to 1% by weight of cement. The concrete specimens were cast into standard prism molds (25 mm × 25 mm × 280 mm) and cured for 28 days at 23 ± 2 °C. The inclusion of GO significantly enhanced the performance, with up to 40% less weight loss and reduced surface damage after 378 freeze–thaw cycles compared to the control. GO also increased the air content by 40% and reduced the mesopore volume, leading to higher compressive strength and improved overall durability. This makes GO a highly effective additive for enhancing concrete’s resistance and strength.

In the study by Zhengyao Qu, Shuaiqi Guo, et al. [28], materials were meticulously prepared to evaluate the freeze–thaw resistance of concrete. A saturated Ca(OH)_2_ solution was created to simulate the alkaline environment of the cement pores, with atactic Polyvinyl Alcohol (PVA) added at a specific concentration to inhibit ice recrystallization. Sucrose was also used in specific assays to further explore this effect. Mortar specimens were prepared using CEM III/A 52.5 N cement, standard sand, and controlled water-to-cement ratios, with PVA included in some of the mixes. The PVA was dissolved in water at precise ratios before mixing to ensure uniformity. The specimens were cast, cured, and tested for the flexural and compressive strengths. The study found that the PVA-modified concrete exhibited a significant reduction in surface scaling—up to 50% less—during freeze–thaw cycles, underscoring PVA’s potential to enhance concrete durability in freezing conditions.

The research conducted by Wei-Wen Li et al. [29] investigated the effect of adding multi-walled carbon nanotubes (MWCNTs) into cement-based materials in order to enhance their mechanical properties and durability. At the very beginning, the MWCNTs were milled with cement at a 40:1 ball/mixture ratio using a QM-QX2 ball mill for 0.5 h. Afterwards, a homogeneous mixture was added to sand, superplasticizer, and water in the cement mixer to obtain fresh Carbon Nanotube (CNT)/cement composites. Proportions of MWCNTs were varied as follows: the addition of MWCNTs was at 0.1 g, 0.3 g, and 0.5 g per 100 g of cement for the Q1-CNT, Q3-CNT, and Q5-CNT specimens, respectively. The specimens were cast into two sets of molds with two different sizes for testing the drying shrinkage, mechanical properties, and microstructure, and then cured either under drying (20 °C; 50% RH) or standard curing conditions. In the freeze–thaw cycling test, 16 h at −20 °C was followed by 8 h in water at 20 °C. The testing for compressive strength was performed after 30, 60, and 90 cycles. The results indicated that the Q3-CNT specimens had far less degradation in the compressive strength than the control specimens, therefore demonstrating better resistance to the freeze–thaw effect. The addition of MWCNTs improved the mechanical properties, which include the compressive and flexural strengths, reduced the extent of drying shrinkage, and improved the pore structure of the composites by reducing micro-crack formation. The MWCNTs’ bridging effect improved the interconnection of hydration products, hence enhancing the overall durability and improving the micro-cracking resistance.

Jiří Šál [30], in a study with coauthors, tested the microstructure, strength and durability, and biomass ash (BA) of cement composites as a partial replacement for Portland cement. The BA used in this research was purchased from one heating plant in the Czech Republic, where it was produced by the combustion of wood chips. The chemical and phase composition of the BA was determined by X-ray fluorescence spectroscopy and X-ray diffraction. Mortar batches were prepared with different biomass ash (BA) contents, from 10% to 70% by mass of Portland Cement (PC), and the mix’s water/binder ratio (w/b) was carefully designed to result in matching a consistent stability, as controlled by the spread flow table test. The ČSN EN 197-1 mixture design guideline was followed in the study. The microstructure and porosity were evaluated by a number of methods in this study, such as SEM, bulk density measurements, and Mercury Intrusion Porosimetry (MIP). The freeze–thaw resistance was tested on cycles of freezing at −20 °C and thawing at +20 °C; the mass loss was measured as an indication of the deterioration. The results showed that the extent of water absorption varied with the percentage of the BA replacement, which thus enabled insights into the potential of BA as an SCM towards improving freeze–thaw resistance.

In the study by Hao L et al. [31], ordinary Portland cement (OPC), sulfoaluminate cement (SAC), and an iron-rich phosphoaluminate cement had the following chemical compositions, wherein an iron-rich Poly-Aluminum Chloride (PAC) clinker was prepared inhouse from calcium phosphate, aluminum oxide, calcium carbonate, iron (III) oxide, and silicon dioxide. This type of cement, with a high early strength and a low heat of hydration, coupled with excellent chemical attack resistance, was evaluated with the performance of SCMs like class fly ash (FA), gypsum (GP), and limestone powder (LP). The size distributions of the particles were measured using the laser particle size analyzer, and their pore structures were measured by MIP. The F-T resistance was evaluated by submitting prismatic specimens to 150–200 cycles of F-T, with periodic measurements for the mass and elastic modulus through transverse frequency tests. The results indicated that iron-rich Poly-Aluminum Chloride (PAC) mortars had a higher resistance to freeze–thaw cycles and smaller pore sizes compared to OPC and sulfoaluminate cement (SAC), which enhanced their durability against hydrostatic pressure. The findings of this study also made clear that, regarding the compressive strength and F-T resistance, the blended systems containing LP outperformed those containing FA and GP. This might underline that it is the role of the pore structure and material composition to provide improved performance in cement-based materials under F-T conditions.

Kumar, P. et al. [32] described the preparation and treatment of materials, among them, the use of ordinary Portland cement, coarse aggregates (CoAs), comprising crushed gravels with a mean size of 11.5 mm, and fine aggregates (FiAs), taken from the bed of the Kaveri River with a fineness modulus of 2.4. The workability of the mix was improved by incorporating a superplasticizer with a water-reducing capacity with a value of 0.26. The ZnO was 99.5% pure, and the individual particle diameter was 85 nm on average. No preconditioning of the nano-ZnO particles was performed before adding them into the mix. These concretes had a constant w/c ratio of 0.41 and were made from four different volume fractions of nano-ZnO particles, namely, 0%, 1%, 2%, and 4%. To obtain the best possible mix, the nano-ZnO particles and the superplasticizer were dispersed in water. The dry ingredients were then added to ensure that the particles were homogeneously distributed. The specimens were cast in prismatic molds (10 cm × 40 cm) and cured under normal conditions after one day at room temperature. The authors calculated that the addition of 2% nano-ZnO to the concrete possessed the highest Comparative Dynamic Modulus (CDM) value and, therefore, demonstrated the highest individual freeze–thaw resistance. The analysis of the measurement with more than 2% nano-ZnO showed a decrease in the CDM value; a high dosage of nano-ZnO may have been responsible for the particle agglomeration, whereas the strength of the concrete also decreased, which affected the durability against this phenomenon.

In the paper by Jeongsoo, Nam et al. [33], frost resistance in fiber-reinforced cementitious composites (FRCCs) reinforced with Polyvinyl Alcohol (PVA) and polypropylene (PP) fibers. The ingredients used included ordinary Portland cement, Class-F fly ash, microsilica sand, water, and a high-range water-reducing admixture. In this regard, PVA fibers that were 8 and 12 mm in length and 40 μm in diameter, and PP fibers that were 12 mm in length and 42.6 μm in diameter, were used to make the FRCC mixtures. Mix proportioning and preparation of the concrete specimens were carried out, and the specimens were exposed to temperature changes from 5 °C to −18 °C, simulating 300 freeze–thaw cycles over a period of 4-h intervals. The study assessed various characteristics related to the freeze–thaw durability, including the compressive and flexural performance, mass loss, and relative dynamic elastic modulus. The results showed that the PVA FRCC specimens exhibited superior resistance to compressive strength reduction compared to the PP FRCC and plain specimens, maintaining durability after the freeze–thaw cycling. These findings underscore the effectiveness of PVA fibers in enhancing frost resistance, highlighting their potential for durable infrastructure in cold regions.

The study by Ming-Gin Lee et al. [34] investigated the effects of replacing cement with recycled diatomite in concrete mixtures. The materials used included recycled diatomite (replacing 10%, 15%, 20%, 30%, and 40% of the cement by weight), silica fume, selected sand, and a high-efficiency water reducer (Chupol SSP-104, with an over 20% water-reduction capability). The mix designs varied, with cement paste (C) containing only cement and water at a 0.6 water–binder ratio, and cement mortar (CM) including cement, sand, and no diatomite or silica fume. Mixtures such as CMS20 (20% diatomite replacement) were tested to evaluate the concrete performance. The freeze–thaw resistance was assessed by subjecting the cement mortar specimens to repeated cycles; the results showed increased weight loss and shrinkage with a higher diatomite content, particularly after multiple freeze–thaw cycles.

The study by Cihang Huang et al. [35] explored the effects of colloidal nano-silica (CNS) on concrete’s freeze–thaw resistance. Concrete samples were prepared by first premixing dry ingredients, and then adding water with an air-entraining agent (AEA) and dispersing CNS into the remaining water. The reference mix contained 335 kg/m^3^ of OPC, with CNS added at 0.3%, 0.6%, and 1.0% of the mix weight. The compressive strength was tested at 7, 14, and 28 days. The freeze–thaw resistance was assessed by measuring the dynamic Young’s modulus. The results showed that 0.6% CNS was the most effective, reducing the void diameter by 25% and increasing the smaller pore percentages, leading to better impermeability and freeze–thaw resistance. However, at 1.0% CNS, the performance declined due to overagglomeration. The study concluded that CNS could enhance the freeze–thaw resistance without needing to increase the AEA, which could otherwise negatively impact the concrete strength.

The study by Ji Yuan et al. [36] examined the impact of wrapping expanded polystyrene (EPS) beads with different materials—Magnesium Phosphate Cement (MPC), Ultra-High-Performance Concrete (UHPC), and Waterborne Polyurethane (WPU)—on the mechanical properties and durability of EPS concrete under freeze–thaw conditions. The high-quality wrapping materials, including MPC with 60–70% magnesium oxide, ensured the effective wrapping and bonding of the EPS beads. These wrapped beads were then mixed with ordinary Portland cement (OPC), sand, and silica fume in a concrete mix with a water-to-cement ratio of 0.45. The study subjected the EPS concrete specimens to 275 freeze–thaw cycles. The results showed no significant surface changes after 175 cycles, but, after 275 cycles, some mortar peeling and EPS bead exposure were noted. The dynamic elastic modulus of the EPS concrete decreased, then increased, and finally dropped to 60% of its initial value, indicating a unique freeze–thaw damage pattern compared to ordinary concrete. Mass loss primarily occurred due to mortar peeling rather than EPS bead loss, demonstrating that the wrapped EPS beads enhanced the concrete’s overall durability.

In the research of Fangyuan Dong et al. [37], they focused on hybrid fiber-reinforced concrete (FRC), with the main binders being subjected to freeze–thaw cycles, including the PII 52.5 cement. Silica fume, fly ash, the other ingredients, and the grade of PE and steel fibers used as reinforcement were kept the same in the concrete mixes for the aforementioned research. Mix proportions were accurate to the amount of cement, which was at 666 g/L, silica fume at 133.2 g/L, and fly ash at 437.7 g/L, while the water-to-binder ratio remained at 0.143. The content of fibers differed in each mix, whereby the PE fiber ranged from 0 to 156 g/L, and the steel fiber ranged from 0 to 117 g/L. The freeze–thaw test involved 250 cycles on concrete specimens cured for 28 days. The result showed that, although the cycles affected the compressive and flexural strengths a little, a decrease was observed in the tensile strain capacity, and in the number of cracks and in the crack spacing. Before carrying out the freeze–thaw tests, the FRC had a high strength improvement over the plain concrete. After adding the fibers, the variation ranged from 31.9% up to 84.0% for the compressive strengths, depending on the fiber contents. The PE fibers gave improved deformability and lower brittleness; yet, this lowering was weak in the fiber/matrix adhesion due to the freeze–thaw cycles, thus causing the improved deformity. The relative dynamic modulus of elasticity indicated a stable compactness of the concrete matrix; therefore, the whole strength remained despite the freeze–thaw effects.

The study of Fatma Karagöl et al. [38] examined the influence of the addition of calcium nitrate as an antifreeze admixture to concrete mixes containing ASTM Type I Portland cement (CEM I 42.5 R). Calcium nitrate was added at 6% by mass of the cement, provided by Tekkim Kimya. A superplasticizer was also used in the concrete at 0.5% to improve the concrete mix’s workability without air entrainment. The aggregates were carefully proportioned according to size fractions to ensure the proper grading of the concrete. The concrete samples were frozen at temperatures of −5, −10, −15, and −20 °C for 7, 14, and 28 days, respectively, and thereafter underwent water curing for the corresponding durations. The results obtained showed that, for the samples with calcium nitrate added, C1, the compressive strengths under freezing conditions ranged from 6.55 to 7.92 MPa. The samples without the calcium nitrate additions had considerably higher values, with results between 15.53 and 35.93 MPa. The results showed that calcium nitrate worked well to avoid drastic compressive strength losses from freezing, with the C1 samples obtaining an 80% reduction in strength at −5 °C compared to the other samples, which proved that this admixture was effective in improving concrete durability under cold climate conditions.

The study by Wei Sun et al. [39] utilized 52.5R (II) Portland cement, river sand (with a fineness modulus of 2.36), and crushed basalt stone (with a maximum size 10 mm) as the coarse aggregate, with steel fibers (at a 20 mm length and an aspect ratio of 40) added to some of the mixes. The concrete specimens were prepared following ASTM C666A standards, subjected to freeze–thaw cycles, and tested in both water and a 3.5% NaCl solution. The concrete mixes varied, with C40NPC having a water-to-cement (w/c) ratio of 0.44, C60NPC at 0.32, and C80NPC at 0.26. Steel fiber-reinforced versions (C40SFRC, C60SFRC, and C80SFRC) included 1.5% steel fiber. The results showed that the concrete exposed to freeze–thaw cycles in the NaCl solution suffered significantly more scaling and mass loss, with the weight loss twice as much as in the water. The freeze–thaw durability of the concrete was lower under higher stress ratios, and the performance deteriorated rapidly. Notably, in the NaCl solution, the dynamic modulus of elasticity decreased more slowly than in the water due to the solution’s lower freezing point. However, the performance degradation was accelerated under combined stress and freeze–thaw conditions. The inclusion of steel fibers significantly improved the resistance to these multi-damaging processes, effectively retarding the performance degradation under severe conditions.

The study by Maciej Gruszczyński and Małgorzata Lenart [40] focused on enhancing concrete mortar by using CEM I 42.5N-NA Portland cement with 5% amorphous aluminum silicate (AAS) and 10% silica fume (SF) as additives. The mix had a water–cement ratio of 0.5 and included quartz sand as the aggregate, with a 1:3 cement-to-sand ratio, and a Rheology-Modifying Admixture (RMA) to achieve a 175 mm slump. The freeze–thaw tests showed that the unmodified mortar lost 10.2% of its weight, while the AAS- and SF-modified mortars lost only 0.1% to 0.2%. Additionally, the AAS improved the flexural strength by 25% to 30% and reduced the shrinkage strain, outperforming the SF, which only increased the strength by 9% to 15%. Both of the additives enhanced the compressive strength, with the AAS also reducing the water demand, thus demonstrating its significant potential in improving the mortar durability and mechanical properties.

**Figure 3:**
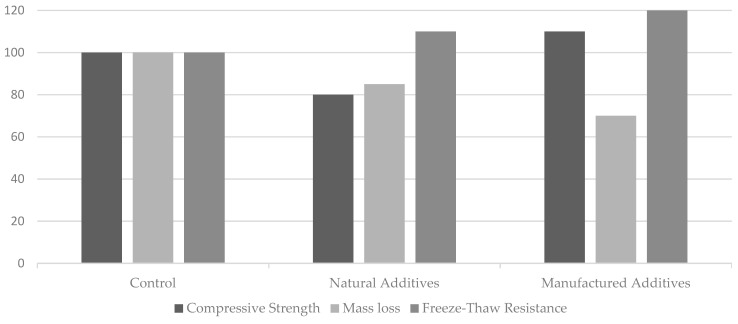
Comparison of the performance of the control (no additives), natural additives, and manufactured additives to F-T cycles.

If we make a comparison between all of these additives and divide them into two groups, manufactured additives and natural additives (see Table 2), we will notice that the manufactured additives generally have a better impact on the concrete resistance to freeze–thaw cycles compared to the natural additives. While both types of additives enhance the freeze–thaw durability, manufactured additives, like nano-SiO_2_/nano-TiO_2_, graphene oxide, silica fume, latex, etc., are particularly noted for their superior performance in improving concrete’s durability against freeze–thaw cycles. These additives tend to offer higher resistance, reduced mass loss, and less internal damage during the freeze–thaw process compared to natural additives. The graph below illustrates the comparative performance of the following three different concrete mix types: control (no additives), natural additives, and manufactured additives (see Figure 3). The performance was assessed across the following three key aspects: the compressive strength, mass loss, and freeze–thaw resistance. The control mix served as the baseline for comparison. Natural additives, which include materials such as zeolite and fly ash, etc., generally improved the durability by reducing the mass loss and enhancing the freeze–thaw resistance (see Table 3), though they may slightly reduce the compressive strength. All of these data were gathered from the literature.

## 3. Investigative Approaches for Specimens

Continuum mechanics deals with solids and fluids, and the effects of the forces on them, divided into general principles for all materials and constitutive equations for specific materials and deformation processes. General principles, such as the conservation of mass, linear and angular momentum, energy, and entropy inequality, are axiomatic [41]. This study modeled the constitutive relations of fly ash concrete under freeze–thaw cycles using Continuum Damage Mechanics (CDM), a framework based on irreversible thermodynamics. A multiple sharp degradation point model was proposed to describe the nonlinear deterioration of concrete’s mechanical properties, particularly the strength and elastic modulus. The applicability of CDM was tested for the stress–strain relations of fly ash concrete after freeze–thaw cycles, confirming its effectiveness and flexibility in durability modeling. CDM enabled the authors to develop comprehensive models for damage and mechanical performance of fly ash concrete under freeze–thaw conditions.

Constitutive models illustrate material reactions under mechanical or thermal loading, helping to formulate governing equations, conservation laws, and kinematic relations through stress–strain relationships [42]. In this study, constitutive models were used to analyze and predict the mechanical behavior of fly ash concrete under freeze–thaw cycles. A damage constitutive model, based on damage mechanics and a multiple sharp degradation point model, was developed to capture the degradation of the compressive strength and elastic modulus during freeze–thaw cycles. This model evaluated the stress–strain relationship of concrete specimens after varying freeze–thaw cycles, accurately predicting the property changes. Its effectiveness was validated against experimental data, showing lower average errors than existing models like Zou and Duan. Derived from physical principles, the model’s parameters enhanced the interpretability and applicability for future research. Its adaptability to other concrete types underscores its versatility in durability studies. Overall, the constitutive models method provided a robust framework for predicting fly ash concrete’s mechanical behavior under freeze–thaw conditions, offering insights into its durability and performance.

Digital Image Correlation (DIC) is a technique that compares digital photographs of a test piece at different deformation stages to measure the surface displacement and develop full-field 2D and 3D deformation vector fields and strain maps by tracking pixel blocks. Effective DIC requires pixel blocks with random variations in contrast and intensity. Often, DIC works without special surface preparation, utilizing the natural texture of the structure or component [43]. In this study, DIC was used to estimate the strain fields during impact failure for basalt fiber-reinforced concrete specimens. The process involved setup, calibration, and imaging using two 2-million-pixel CCD cameras during impact tests. A speckle pattern applied to the specimens improved the correlation accuracy, enabling better displacement tracking. Images recorded at each impact were processed using DIC software (vic-3D) to determine the displacements and strains. DIC allowed for the real-time observation of the strain distribution, thus clarifying the damage evolution under impact loading. The displacement accuracy was evaluated using a correlation coefficient, with the X- and Y-direction displacements quantified to provide a comprehensive view of the strain changes. The results showed that the 3D space system created by basalt fibers extended the impact stress wave transmission, thereby enhancing the concrete resistance. DIC also revealed the fiber rupture and degradation of the fiber/matrix interface after freeze–thaw cycles, thus reducing the fiber reinforcement. Overall, DIC provided valuable insights into the mechanical behavior of basalt fiber-reinforced concrete under dynamic loading.

Scanning electron microscopy (SEM) is a powerful tool for investigating micro- and nano-scale structures. It creates images by emitting high-energy electrons, with the voltage settings affecting the details obtained: a low voltage provides surface information, while a high voltage penetrates deeper. The image quality depends on visualizing the sample topography, with surface inclination angles enhancing Backscattered Electron (BSE) and Secondary Electron (SE) signals, thus improving the topographic contrast [44]. SEM is effective for analyzing the microstructure of cement and concrete, evaluating the impact of supplementary materials on the durability, and predicting the service life of concrete structures [45]. In this study, SEM was used to examine the microscopic morphology of UHMWPE-ECC specimens subjected to freeze–thaw cycles and carbonation. SEM provided insights into the surface damage and structural changes, clarifying uncertainties about minor surface damage and sub-micrometer crack formation due to freeze–thaw cycles. It revealed fiber–matrix bonding and showed that carbonation roughened the fiber surfaces, thereby enhancing their interaction with the cement matrix. which is crucial for the composite’s mechanical performance. SEM also indicated that freeze–thaw cycles loosened the matrix around the fibers, thereby reducing the compressive strength and tensile strain. Overall, SEM played a vital role in understanding the material behavior under environmental conditions, aiding in assessing the durability and performance for practical applications.

Industrial computed tomography (CT), also known as industrial CT scanning or 3D X-ray scanning, is a non-destructive technique used to generate high-accuracy 3D models of an object’s internal structure by rotating the object and capturing X-ray images from multiple angles, which are reconstructed using algorithms to enable the precise inspection of internal features like the material properties, voids, and defects. Widely applied in aerospace, automotive, electronics, and materials industries for quality control, failure analysis, and research [46], industrial CT offers advantages such as the high-resolution, non-destructive imaging of internal and external features without dismantling [47]. In this study, the CT method was applied to investigate the internal structure and deterioration of concrete specimens under freeze–thaw cycles using the Multiscale Voxel 450 system, equipped with a high-power X-ray source, high-sensitivity detector, and precise motion control for high spatial resolution. Concrete specimens with varying nano-SiO_2_ and nano-TiO_2_ contents were prepared, subjected to freeze–thaw cycles, and scanned with adjusted current and voltage settings, producing detailed sectional projection images, processed using Avizo software to visualize the pores, cracks, and internal deterioration. The CT images revealed the impact of the nanoparticle additions on the concrete’s internal integrity, comparing the specimens with nano-additives to normal mixes so to assess their effectiveness in enhancing freeze–thaw resistance and reducing internal damage, thereby demonstrating industrial CT’s essential role in visualizing and analyzing internal deterioration and evaluating the influence of nanoparticle additions on concrete performance under freeze–thaw conditions.

Unconfined compressive strength (UCS) is a critical property in rock engineering, representing a fundamental challenge in estimating reliable values from highly variable data. The method aims to determine the minimum number of cores needed to achieve a reliable average strength estimate and to assess the variability of UCS in a given rock type. Statistical tests on approximately 50 core samples from five rock types revealed that the minimum sample size depends on the chosen confidence interval, statistical technique, and accepted standard deviation from the mean, with 9 or 10 samples required for a 95% confidence interval and a 20% acceptable strength deviation [48]. In this research, the UCS method was used to evaluate the strength properties of soil specimens with varying cement and polypropylene (PP) fiber contents. Soil samples were prepared, cured for seven days, and tested using the Material Testing Systems (MTS-810) apparatus, following the Specification of Soil Tests JTG E51-2009, under constant displacement rates of 2 mm/min until failure or 8% axial strain. Axial displacement and vertical load were recorded at five points per second, enabling the construction of stress–strain curves to analyze the mechanical behavior. Peak stress, identified as the UCS, represented the maximum load before failure under unconfined conditions. The UCS values were compared across freeze–thaw cycles and varying cement and fiber contents, with the results presented in tables and figures to clarify the effects of these variables on the soil strength. The UCS method thus involves systematic specimen preparation, testing, data recording, and analysis to evaluate the strength of treated soils under unconfined conditions.

Empirical equations provide a reliable foundation for design codes to estimate mechanical concrete properties, with ongoing experiments aimed at improving the estimation of the modulus of elasticity (MOE) by considering factors like the aggregate type, paste composition, mix design, and concrete density [49]. The need for experimental data to correlate empirical models, based on mass and heat transfer mechanisms, remains critical [50]. In this research, empirical models were used to estimate the unconfined compressive strength (UCS) of stabilized clay, focusing on key influencing factors such as the cement content, fiber content, and freeze–thaw cycles. A multivariate nonlinear regression analysis was employed to develop a mathematical model relating the UCS to these factors, with regression coefficients determined from the UCS test data under varying conditions. The model’s accuracy was validated by comparing the predicted results with the experimental data, showing a strong agreement and effectively capturing the relationships between the variables. This validated model serves as a practical tool for predicting the UCS in stabilized clay under different conditions, offering valuable insights for soil stabilization techniques, particularly in cold regions, and systematically explaining the strength behavior in characterized stabilized clay soils.

The remediation of waste is crucial in concrete production to prevent material wastage during mixing, pouring, and curing. Construction activities generate significant amounts of scrapped concrete, known as “concrete rubble” or “recycled concrete aggregate” (RCA). The use of supplementary cementitious materials like fly ash and slag promotes sustainable concrete practices, reducing material wastage and environmental impact while recycling concrete waste for greener construction [51,52]. This study adopted a waste mitigation approach by developing hemp and recycled aggregate concrete (HRAC), replacing 50% of the natural coarse aggregates with RCA to reduce the resource demand and address construction waste issues. Additionally, 20% of the fine aggregate was replaced with sustainable hemp fibers, which enhanced the mechanical strength while adhering to sustainability guidelines. This approach highlights the application of recycled aggregates and natural fibers in reducing waste and promoting sustainable construction practices.

Full-field strain measurements are essential for testing textile deformability during composite processing, serving the following three primary purposes: (1) acting as an “optical extensometer” to accurately measure sample deformation during shear and tensile tests, which may differ from the applied deformation; (2) studying textile deformation mechanisms at the unit cell and yarn level, providing insights into meso- and micro-scale deformations; and (3) capturing the 3D-deformed shape and local deformation distribution of textile reinforcements after draping, enabling the validation of draping models and the prediction of consolidated part performance via structural finite element analysis. Optical full-field strain techniques, such as Digital Image Correlation (DIC), are critical for accurate deformation measurements in woven (e.g., glass and glass/PP) and non-crimp (e.g., carbon) textile reinforcements [53]. In this study, the full-field strain method, utilizing DIC technology, was employed to measure and analyze the strain distribution across concrete specimens during impact testing. Specimens with varying basalt fiber contents were prepared, and their surfaces were treated with a speckle pattern for precise displacement tracking. During the impact testing, a ball was dropped from a set height, and images were captured before and after each impact using high-resolution CCD cameras connected to a DIC system. The DIC software (vic-3D) processed these images to calculate the displacements and generate detailed strain distribution maps, highlighting the areas of maximum strain, such as crack tips. This enabled the real-time monitoring of crack initiation and propagation, revealing the effects of impact loads and basalt fibers on the damage process. The results demonstrated how basalt fibers enhanced the mechanical performance and how freeze–thaw cycles affected the concrete integrity. The DIC-based full-field strain method provided valuable insights into the strain distribution, crack formation, and impact resistance of basalt fiber-reinforced concrete under dynamic loading conditions.

Various ASTM International standards outlined include the following critical tests for some properties of concrete, which were used in various mentioned papers. Standard Test Method for Slump of Hydraulic-Cement Concrete gives the workability of concrete as measured by its slump, which will assure it is suitably placed and compacted. The Standard Test Method for Compressive Strength shows the strength of concrete by axially compressing specimens, which is very important in quality control and mix optimization. Other tests, like the modulus of elasticity, splitting tensile strength, and thermal transmission properties, give basic data for structural sizing, reinforcement determination, and the assessment of thermal conductivity. Standardized procedures have an important role in ensuring concrete structure quality, performance, and durability for works on construction and engineering [54,55,56,57,58,59,60,61,62].

Looking back at the sophisticated analysis carried out in this section, one does not need to mention that the application of various advanced techniques, particularly scanning electron microscopy and testing for the unconfined compressive strength, provided striking insights into the material behavior under diversified environmental conditions. The empirical models developed herein were validated and found to be powerful tools in predicting the material performance. While these methods have had some success in pointing out a number of relevant variable interactions, additional studies will be necessary to take into account other factors that might mediate the material properties. As this section points out, what is really required is an interplay among the experimental data and predictive modeling to further improve our understanding of the durability and engineer practices in the future.

All investigative approaches described above are synthesized in Table 4.

## 4. Impact of SCMs on Concrete Under Freeze–Thaw

Experimental evidence has shown that freeze–thaw cycles drastically reduce the elastic deformation capacity and impact strength of concrete, with increased cycles being abundant in increased damage. This indicates the importance associated with the consideration of the environmental factors in the design of concrete. Various kinds of materials and methods have been extensively studied for enhancing the performance of concrete against these cycles, which has led to increasing the durability and lifespan of service.

**Figure 4:**
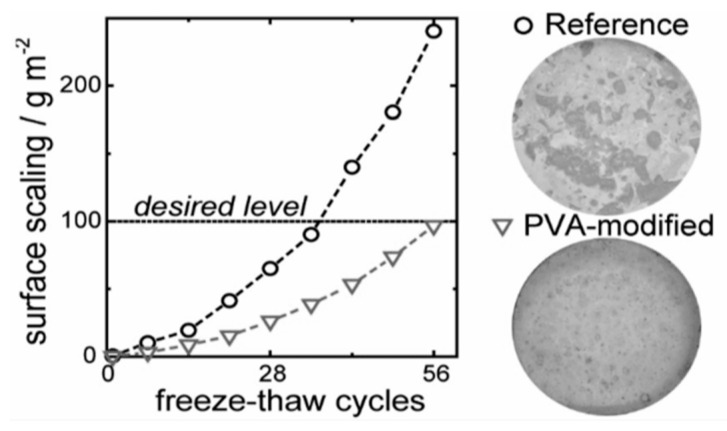
Cumulative surface scaling of a concrete mix with PVA after F-T cycles [28].

Additives to the material, such as graphene oxide (GO) and Polyvinyl Alcohol (PVA), have been demonstrated to improve the freeze–thaw resistance. Samples with GO exhibited less weight loss and lower damage compared to the control mixes, whereas the concretes modulated with PVA significantly reduced the surface scaling (see Figure 4). These observations may indicate that such additives can be effectively used for the mitigation of the adverse effects of freeze–thaw cycles.

**Figure 5:**
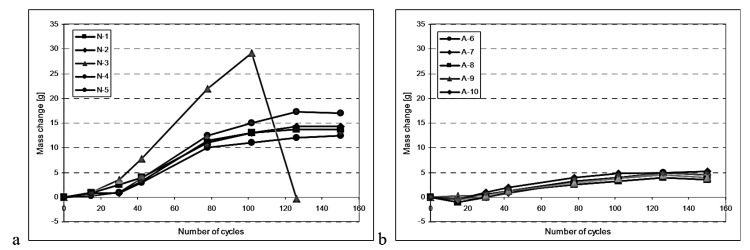
Mass change in concrete: (**a**) non-air-entrained GGBS specimens; (**b**) air-entrained GGBS specimens [18].

Other alternative cementitious materials also lead to increased freeze–thaw resistance. Studies on using GGBS (Figure 5) and biomass ash (BA) as partial replacements for Portland cement have shown beneficial effects. Although GGBS in certain percentages reduces the permeability and improves the durability, BA is effective at some percentages in the mix and requires much care in its use.

**Figure 6:**
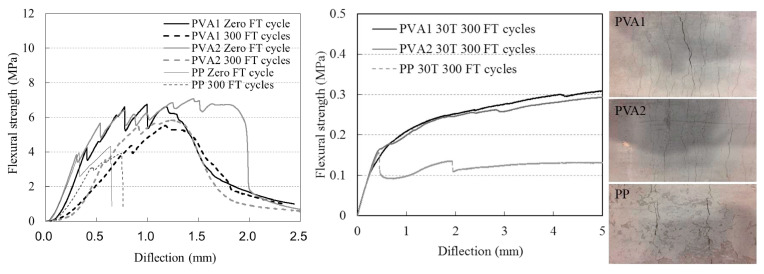
Flexural response and failure of the FRCC specimens under F-T [33].

Fiber-reinforced cementitious composites (FRCCs) show a small reduction in the compressive and flexural strengths after freeze–thaw cycles (Figure 6), where a huge reduction in the deformability and cracking behavior was observed. However, the deformability was higher and the brittle failure was lower because of the inclusion of polyethylene (PE) fibers. All of these explained well the concrete performance with hybrid fiber reinforcement under freeze–thaw conditions.

**Figure 7:**
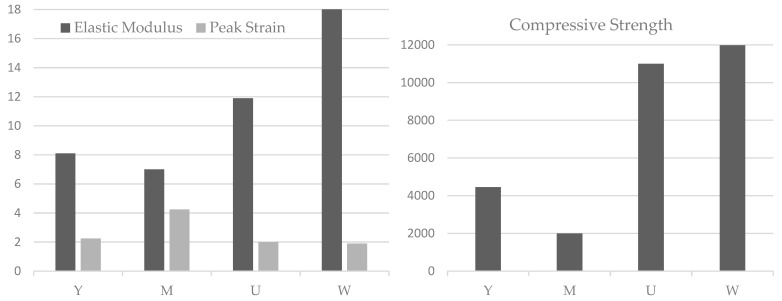
EPS concrete compression performance after 250 F-T cycles, where Y, M, U, W are different groups of relative dynamic elastic modulus attenuation [36].

The freeze–thaw resistance of geopolymer concretes is excellent, showing a less than 5% mass loss even after extensive cycles. This can be attributed to its lower porosity and the further beneficial actions of pozzolans like metakaolin and silica fume, thus contributing to the improved durability. Similarly, expanded polystyrene (EPS) concrete portrayed a particular freeze–thaw damage pattern. In this case, the mass loss was primarily caused by the peeling of loose mortar rather than by the loss of the EPS beads, stress–strain, and compressive tests (Figure 7). The positive contribution of the EPS beads to the durability of the concrete can, therefore, be affirmed.

**Figure 8:**
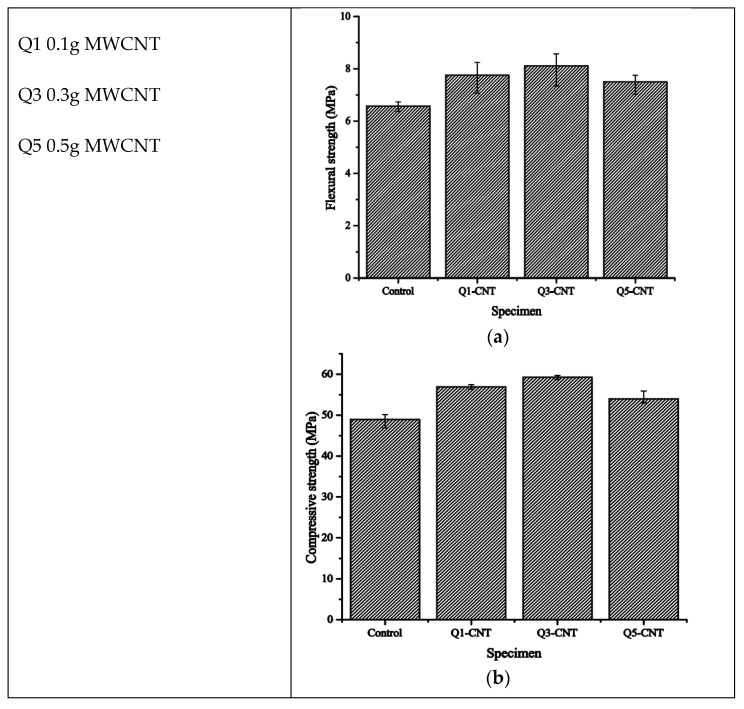
Flexural (**a**) and compressive strengths (**b**) of the MWCNT specimens after 28d of standard curing [29].

**Figure 9:**
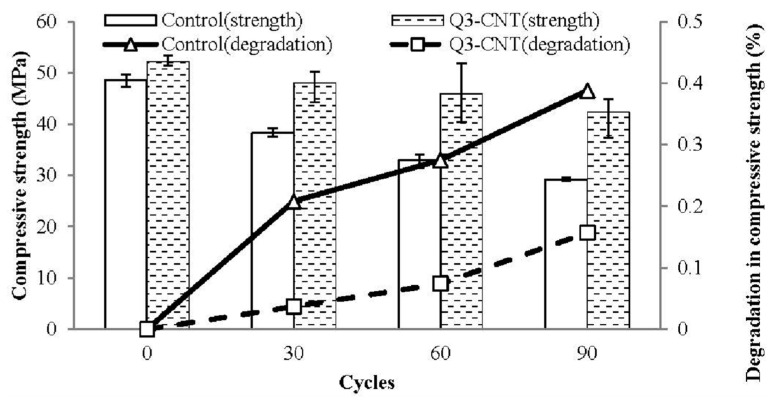
Degradation in compressive strength of the control and Q3-CNT specimens under freeze-thaw (FT) cycles [29].

Nanomaterials, such as multi-walled carbon nanotubes (MWCNTs), have also been investigated for their potential to enhance the freeze–thaw resistance. Specimens incorporating MWCNTs demonstrated better resistance to freeze–thaw-induced degradation compared to the control specimens, highlighting the potential of nanomaterials in improving the durability of cement-based materials, as the addition of MWCNTs led to an increase in the flexural and compressive strength, and, as shown in Figure 8 and Figure 9, the decrease in the compressive strength for Q3-CNT was lower than that of the control, indicating a better resistance of the CNT/cement composite against F-T.

Therefore, the compactness of the concrete matrix is significant in relation to resistance to freezing and thawing, as evaluated with the relative dynamic modulus of elasticity. A closer compaction of the matrix to the maximum level relates to the better retention of the strength in the face of freezing–thawing effects, thus proving that keeping the integrity of the matrix is a top condition for long-term durability.

The freeze–thaw cycles in terms of the cracking behavior induce changes characterized by a reduction in the capacity of the tensile strain, the number of cracks, and the spacing. These changes depend on the concrete composition and environmental conditions; hence, the mechanisms of damage can be shown to be complex, with multiple factors at play.

**Figure 10:**
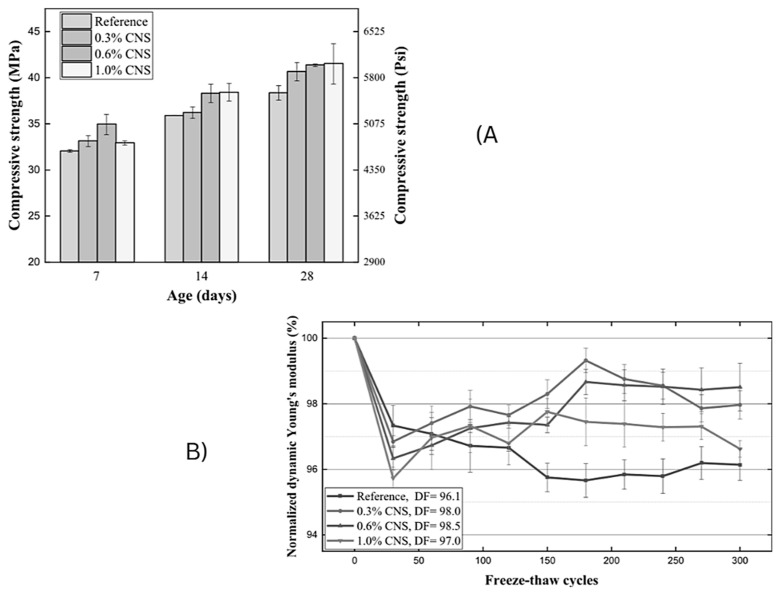
CNT specimen in (**A**) the compressive test result; (**B**) the development of the normalized dynamic Young’s modulus under F-T cycles [35].

The effectiveness of additives like Calcium Nitrite, Ca(NO_3_), is dosage-dependent. A specific dosage of 0.6% provided the best freeze–thaw resistance, while higher dosages led to performance deterioration due to overagglomeration. This underscores the importance of carefully optimizing the additive concentrations so to achieve the desired durability outcomes. According to the compressive test results (Figure 10), as the Ca(NO_3_) doses increased from 0% to 1.0%, the compressive strength improved by 6%, 7.8%, and 8.2%. The primary causes of this improvement were the pozzolanic reaction of Ca(NO_3_) and the enhanced hydration facilitated by the nanoparticles.

### Concrete Service Life

The service life of concrete is a major factor when it comes to infrastructure sustainability and durability. F-T cycles are one of the many factors that affect the life of concrete, and the concrete service life is closely related to environmental resistive actions such as freeze–thaw cycles, chemical attack, and mechanical wear. Prolonging the service life of concrete decreases the frequency of repair and replacement, thus reducing the environmental impacts related to the production and transport of concrete materials, and reduced CO_2_ emissions, which leads to a more sustainable environment. Among the identified advantages of SCMs is their potential to diminish the global warming potential due to the use of cement-based materials [63,64,65].

As discussed by Mackechnie and Alexander, an improved concrete durability performance increases the service life through an enhanced structural capacity, with smaller deformations under service conditions. This provides the possibility of designing lighter and more stable structures with less conservative safety margins. Indeed, high-performance concrete materials with dense matrices, which are resistant to crack formation, exhibit better durability and mechanical properties, thus extending the service life of these concrete structures [66,67].

SCMs thus represent an important avenue for enhancing the concrete durability, such as fly ash, silica fume, and GGBS. These supplementary materials contribute considerably toward the reduction in the environmental impact of concrete production by replacing a certain part of Portland cement and, at the same time, enhancing the material resistance to environmental stresses. For instance, fly ash and silica fume increase the density of the microstructure, which further decreases the permeability and gives better resistance to freeze–thaw cycles, which is a major concern in enhancing the lifespan of concrete in cold climates [68,69,70].

Advanced materials such as graphene oxide, nano-silica, and synthetic fibers have also been investigated for the incorporation into concrete, with the intention of further enhancing its durability and service life. Each of these materials enhances the properties of concrete in aspects that are critical toward ensuring that it stays structurally sound, and all are related to the sustainability of concrete structures. The ability of a structure to remain functional, without major maintenance or replacement for a longer period, means a reduced overall environmental impact. Meeting the needs and concerns about improving the concrete durability and service life will make for more sustainable construction. This also aligns with the principles of SBE, including resource conservation, life cycle costing, and human-friendly design [64,71,72].

The environmental benefits from this extended concrete service life will likewise be quite significant. Noticeably reduced repair and replacement translate into much-reduced demands for both raw materials and energy, and, conversely, actually lower the associated greenhouse gas emissions owing to the production and transport of cement. Designing concrete for a longer service life will also be consistent with resource efficiency and therefore contribute to general sustainability objectives [71,73,74]. The F-T and durability characteristics of concrete can easily be affected and lead to the failure of the structure. Improving the concrete and gaining a healthier environment is a great thing to achieve. The production of cement causes the generation of about 5–7% of total anthropogenic CO_2_ emissions. On the other hand, the partial substitution of OPC resulted in a reduction in CO emissions without compromising the mechanical and durability performance of concrete by using all-natural and sustainable additives to the concrete mix [75,76,77,78].

## 5. Conclusions

The review went into detail about the huge strides that have been made toward the application of advanced materials, including geopolymer concretes and pozzolanic additives like metakaolin and silica fume, in enhancing the freeze–thaw resistance of concrete. These work by reducing the porosity and refining the pore structure, hence minimizing water ingress and the resultant freeze–thaw damage. Other innovative additives, however, have also shown promise: acid-leached rice husk ash, synthetic zeolites, and glass particles used to improve the concrete durability through matrix densification, air void reduction, and associated improvements to the mechanical properties. These findings are of direct importance regarding the long-term performance of infrastructures exposed to harsh environmental conditions. The role of nano-technology in enhancing the durability of concrete has been quite imperative; for instance, the incorporation of nanoparticles into concrete, such as nano-ZnO or MWCNTs, has greatly improved its microstructure and overall performance. However, the dosage and proper dispersion are the concerns that need to be taken into consideration in order to avoid possible problems, like the agglomeration of particles, thereby reducing the material integrity. In this regard, special cements—like iron-rich PAC—combined with new additives, like latex and biomass ash, have been underlined to make it possible to develop tailored material formulations respecting specific environmental and mechanical requirements, in particular when exposed to freezing–thawing cycles. Where freeze–thaw conditions exist, geopolymer concrete or pozzolanic additives shall be considered first, since they turn out to show good durability. More advanced additives and materials, such as synthetic zeolites, glass particles, or nanoparticles, can be added to even further enhance the resistance to freeze–thaw cycles, provided that good testing and quality control procedures are followed. The latter consists of latex-modified concrete or special cements that further increase the resistance to scaling and chemical attack under de-icing salts or very aggressive weather, ensuring that what is expected from the response to extreme environments is appropriately tailored. Though the improved performance of these advanced materials are very promising, further studies are required to create a good understanding of their durability and environmental impacts during service. Principally, regarding the use of nanoparticles and biomass-based additives, studying the synergistic effects from combining different materials and additives can unleash new performance and sustainability. Further ahead, standardization and guideline development, considering the newest results in this respect on freeze–thaw-resistant materials, should also be within the sight of the construction industry. It should assist the engineers and builders in better decision making with regard to better materials and their uses. In summation, the latest studies on freeze–thaw-resistant materials for plumbing and construction show wonderful improvements and developments. Geopolymer concrete, with its innovative and sustainable additives, such as natural biomaterials and byproducts, provides better durability, a reduced environmental impact, and higher sustainability than traditional materials. These good practices, and the consideration of future research directions, can confer the construction industry with greater preparedness toward the impacts of freeze–thaw cycles on infrastructure lifespan and mitigate these impacts toward creating more sustainable and resilient built environments.

While this review covered a wide range of materials and testing methodologies, several research gaps remain:

1. Long-term durability studies—More experimental research is needed to assess the real-world performance of SCM-based concrete over extended service periods.
2. Optimization of natural SCMs—Future work should focus on refining the processing and mix proportions of biomass ash, zeolite, and agricultural waste-based SCMs to enhance their F-T resistance.
3. Hybrid additive systems—The combined effects of nanomaterials, fibers, and traditional SCMs should be explored to develop synergistic mix designs.
4. The availability and cost of certain high-performance additives (e.g., graphene oxide and nano-SiO_2_) could limit their widespread adoption in practical applications.

Future research should focus on defining performance-based criteria that consider the mechanical properties to be conducted under standardized conditions so to assess their effects on both the strength and long-term durability, thereby investigating the synergistic effects of multiple additives.

## Figures and Tables

**Table 1:** Comparison of standardized methods and non-standardized methods.

	Standardized Methods	Non-Standardized Methods
Focus	Primarily assess the combined effect of freeze–thaw cycles and de-icing salt exposure. They aim for repeatability and comparability between labs.	Often tailored to investigate specific aspects or mechanisms of freeze–thaw damage with de-icing salts. They may explore the influence of different salt types, concentrations, or environmental conditions.
Advantage	Comparability: Results from different labs can be compared directly. Reproducibility: Standardized procedures minimize the variability. Acceptance: Often required for concrete declarations and specifications.	Flexibility: Can be adapted to investigate specific research questions. In-depth analysis: May provide more detailed insights into the damage mechanisms. Real-world relevance: Field trials can offer valuable information about the performance under actual service conditions.
Disadvantages	Simplification: May not perfectly simulate real-world conditions (e.g., specific salt types, temperature variations, and wetting/drying cycles). Time-consuming: Tests can take weeks or months to complete. Costly: Requires specialized equipment and trained personnel.	Lack of comparability: Results may not be directly comparable between different studies. Variability: Results can be influenced by uncontrolled factors. Limited acceptance: Not typically used for concrete declarations.

**Table 2:** Material comparison data.

Aspect	Control (Base Value)	Natural Additives	Manufactured Additives	Overall Estimate
Compressive Strength	100%	Zeolite: Increases the durability	Calcined Clay: Less than 5% mass loss (high durability)	80% based on the fly ash reduction; offset by zeolite and hemp fiber improvements
		Fly Ash: Compressive strength drops to 49% after 30 cycles	Metakaolin: Less than 4% mass loss; increases the strength	110% based on the significant strength gains from metakaolin and Silica Fume
		Hemp Fiber: Higher relative dynamic modulus of elasticity (RDME), indicating better retention	Silica Fume: Increases the compressive strength by up to 28%	
Mass Loss	100%	Zeolite: Reduces mass loss by more than 1.6 times	Calcined Clay: Less than 5% mass loss after multiple cycles	85% due to reductions by Zeolite, biomass ash, and rice husk ash
		Biomass Ash: Mass loss reduced to 1.3% vs. 1.6% in the control	Metakaolin: Less than 4% mass loss after 300 cycles	70% due to low mass loss (<5%) from calcined clay, metakaolin, and silica fume
		Rice Husk Ash: Significantly lower mass loss than the control	Silica Fume: Less than 5% mass loss	
Freeze–Thaw Resistance	100%	Zeolite: Improves the freeze–thaw resistance up to 3.5 times	Calcined clay, Metakaolin, and Silica Fume: Show significant improvements in the freeze–thaw resistance	110% based on zeolite’s large increase, and contributions from the hemp fiber and rice husk ash
		Hemp Fiber: RDME values up to 90.4% after 144 cycles	Polycarboxylate Superplasticizer: Increases the freeze–thaw resistance by 33.3%	120% due to strong improvements from calcined clay, metakaolin, silica fume, and polycarboxylate superplasticizer
		Rice Husk Ash: Durability Factor (DF) remains above 80% after 300 and 600 cycles		

**Table 3:** Preparation methods in the concrete reported in the literature in Section 2.

References	Material	Replacement Percentage (Weight)	Cement Type	*w*/*c* Ratio	Freeze–Thaw (Cycles)	Curing Duration (Days)
Shinkafi, A. B. (2020) [13]	Calcined clay	20%	CEM-I 52.5 N, geopolymer concrete	0.45	125 and 300	7, 28, and 56
Zhang, S et al. [14]	Metakaolin	9% and 15%	CEM I 42.5R	0.4	300	90
Park, Cheolwoo et al. [15]	Rice husk ash	5% and 10%	ASTM Type I	0.38 and 0.45	300 and 600	14
Park, Cheolwoo et al. [15]	Silica fume	5% and 10%	ASTM Type I	0.38 and 0.45	300 and 600	14
Nagrockiene, Dzigita, and Giedrius Girskas [16]	Zeolite	2.5, 5, 7.5, and 10%	CEM I 42.5R	0.45	219, 519, 546, 632, and 728	28
Ming-hui and Yuan-feng [17]	Fly ash	32%	Ordinary silicate cement	0.5	0, 5, 15, 30, 50, 75, 100, and 125	28
Jerzy Wawrzenczyk et al. [18]	Blast furnace slag	50%	CEM I 42.5 R	0.40–0.50	150	80
Raed M. Abendeh et al. [19]	Recycled glass	2.5, 5, 7.5, 10, 12.5, and 15%	Ordinary Portland cement	0.5	0, 100, 200, and 230	7, 28, and 60
Yutao Bi et al. [20]	Ultra-high-molecular-weight polyethylene	4%	P-II 42.5R Portland cement	0.5	300	1
Shuldyakov et al. [21]	Polycarboxylate superplasticizer	-	Medium alumina cement (CEM I 42.5)	-	300	-
Kyong-Ku Yun et al. [22]	Latex	0%, 5%, 10%, 15%, and 20%	Ordinary Portland cement	0.49, 0.46, 0.42, 0.36/0.38/0.40, and 0.34	300	28
Fang Liu et al. [23]	Nano-SiO_2_/nano-TiO_2_	0.2, 0.4, 0.6, and 0.8%	Ordinary Portland cement 42.5	0.5	25, 50, and 75	28
Dinga et al. [24]	Polypropylene fiber	3%, 6%, 9%, and 12%	CEM II 32.5R	-	0, 1, 5, and 10	7
Samer, G et al. [25]	Hemp fiber	-	Portland cement	0.5	0, 36, 72, 108, and 144	28
Yan-Ru, Z et al. [26]	Basalt fiber	0.0 kg/m^3^, 1.0 kg/m^3^, 1.5 kg/m^3^, 2.0 kg/m^3^, and 2.5 kg/m^3^	-	0.48	15, 30, 45, 60, and 75	120
A. Mohammed et al. [27]	Graphene oxide (GO)	0.01, 0.03, and 0.06%	Ordinary Portland cement	-	540	28
Zhengyao Qu, Shuaiqi Guo, et al. [28]	Polyvinyl Alcohol (PVA)	-	Portland cement	0.5	0, 1, 7, 14, 21, 28, 35, 42, 49, and 56	7 and 28
Wei-Wen Li et al. [29]	Carbon nanotubes (CNT)	0%, 0.1%, 0.3%, and 0.5%	Type I 42.5R Portland cement	0.45	30, 60, and 90	28
Jiří Šál et al. [30]	Biomass ash	10–70%	CEM I 42.5 R	-	0, 50, 100, 150, 200, and 300	28
Hao L et al. [31]	Iron-rich PAC	3–9%	Ordinary Portland cement	-	150–200	-
Kumar, P. et al. [32]	Nano-zinc oxide	0%, 1%, 2%, and 4%	Portland cement	0.41	100	28
Jeongsoo, Nam et al. [33]	Polyvinyl Alcohol (PVA) fiber	-	Ordinary Portland cement	-	300	-
Ming-Gin Lee et al. [34]	Heat-treated diatomite	10%, 15%, 20%, 30%, and 40%	Ordinary Type I cement	-	600	28
Cihang Huang et al. [35]	Colloidal nano-silica (CNS)	0.3, 0.6, and 1%	Ordinary Type I cement	0.42	300	7, 14, and 28
Ji Yuan et al. [36]	Expanded polystyrene (ESP)	-	Ordinary Portland cement (OPC)	0.45	50, 150, 175, and 275	-
Fangyuan Dong et al. [37]	Polyethylene fiber (PE)	0% and 2%	P-II 52.5 R cement	0.4	0, 275	28
Fatma Karagöl et al. [38]	Calcium nitrate	6%	CEM I 42.5 R	0.40	-	7, 14, and 28
Wei Sun et al. [39]	Sodium chloride	-	CEM II 52.5R	0.44, 0.32, and 0.26	500, 1000, and 1500	-
Maciej Gruszczyński and Małgorzata Lenart [40]	Amorphous aluminum silicate (AAS)	5% and 10%	CEM I 42.5N-NA	0.5	25	7, 28, and 90

**Table 4:** Special methods used in investigating F-T in literature.

References	Method	Application	Limitations	Used in
Lai, W. M., Rubin, D., and Krempl, E. [41]	Continuum mechanics	Analyze and predict the principles and behavior of materials.	Inaccurate in scenarios involving large deformations or nonlinear material behavior.	Ming-hui and Yuan-feng [17]
Zhang, X., Chen, Z., and Liu, Y. (2017) [42]	Constitutive models	Describe how materials respond to various loads and environmental conditions, and define the relationship between stress and strain, characterizing the material’s behavior under different types of deformation.	They often rely on idealized assumptions and simplifications, which may not capture the full complexity of real material behavior, particularly under extreme conditions.	Ming-hui and Yuan-feng [17]
Nick, M and Jerry, L [43]	Digital Image Correlation	Non-contact optical method used to measure full-field displacements and strains on the surface.	Accuracy and resolution depend on the quality of the imaging system, the stability, and the lighting, and requires a clear line of sight to the specimen surface.	Yan-Ru, Z et al. [26]
1—Azad, M, and Avin, A [44] 2—Paul E. Stutzman [45]	Scanning electron microscopy (SEM)	Used to examine the surface morphology and composition of a wide range of materials at high magnifications, providing detailed high-resolution images.	Preparation of samples for SEM analysis can be complex, requiring specimens to be conductive or coated with a conductive material.	Yutao Bi et al. [20]
1—Villarraga Gómez, H. (2016) [46] 2—L. De Chiffre et al. [47]	Industrial computed tomography (CT)	Used to create detailed 3D images of the internal and external structures of objects.	The resolution depends on the size of the object and the capabilities of the CT system.	Fang Liu et al. [23]
Richard M. Ruffolo and Abdul Shakoor [48]	Unconfined compressive strength	Measures the maximum axial compressive stress that a material, characterizing the mechanical behavior of materials under compression.	The UCS is a measure of the peak strength and does not provide information about post-failure behavior.	Dinga et al. [24]
1—Behnam, V., and Shami, N. [49] 2—Badr, Bin, A. et al. [50]	Empirical models	Predicts outcomes based on observed data without necessarily understanding the underlying mechanisms, and constructed by analyzing experimental or historical data to establish relationships between input and output variables.	Accuracy and reliability are heavily influenced by the quality and extent of the available data.	Dinga et al. [24]
1—K. Arunkumar [51] 2—C. Kicheek [52]	Mitigation of wastes	Reducing waste at the source through process optimization, using recyclable and biodegradable materials, and using it in the engineering field.	It does not entirely eliminate it, necessitating a continued focus on efficient waste management practices; it requires comprehensive planning, investment, and public cooperation.	Samer, G et al. [25]
S.V.Lomov et al. [53]	Full-field strain	Technique used to capture the distribution of strain across the entire surface of a material or structure under load, providing detailed spatial information on how a material deforms.	Environmental factors such as vibrations and temperature changes can introduce noise and errors into the data; they cannot provide information about subsurface or internal strains, which can be crucial in analyzing the full mechanical response of materials and structures.	Yan-Ru, Z et al. [26]

## Data Availability

No new data were created or analyzed in this study. Data sharing is not applicable to this article.

## Abbreviations

The following abbreviations are used in this manuscript:

AAS	Amorphous Aluminum Silicate
AEA	Air-Entraining Agent
ASTM	American Society for Testing and Materials
BA	Biomass Ash
BSEs	Backscattered Electrons
CCD	Charged-Coupled Device
CDM	Comparative Dynamic Modulus
CEM	Cement (as used in specific grades like CEM I 42.5R)
CM	Cement Mortar
CNS	Colloidal Nano-Silica
CNT	Carbon Nanotube
CoAs	Coarse Aggregates
CT	Computed Tomography
DIC	Digital Image Correlation
ECCs	Engineered Cementitious Composites
EPS	Expanded Polystyrene
FA	Fly Ash
FiAs	Fine Aggregates
FRC	Fiber-Reinforced Concrete
FRCCs	Fiber-Reinforced Cementitious Composites
F-T	Freeze–Thaw
GA	Glass Aggregate
GGBS	Ground Granulated Blast Furnace Slag
GO	Graphene Oxide
GP	Glass Powder
HRAC	Hemp and Recycled Aggregate Concrete
HRWR	High-Range Water Reducer
ISO	International Organization for Standardization
LP	Limestone Powder
MIP	Mercury Intrusion Porosimetry
MOE	Modulus of Elasticity
MPC	Magnesium Phosphate Cement
MSA	Maximum Size Aggregate
MTSs	Material Testing Systems
MWCNTs	Multi-Walled Carbon Nanotubes
OH	Hydroxide
OPC	Ordinary Portland Cement
PAC	Poly-Aluminum Chloride
PC	Portland Cement
PE	Polyethylene
PP	Polypropylene
PS	Polycarboxylate Superplasticizer
PVA	Polyvinyl Alcohol
RCAs	Recycled Concrete Aggregates
RHA	Rice Husk Ash
RMA	Rheology-Modifying Admixture
SAC	Sulfoaluminate Cement
SCMs	Supplementary Cementitious Materials
SEs	Secondary Electrons
SEM	Scanning Electron Microscopy
UCS	Unconfined Compressive Strength
UHMWPE	Ultra-High-Molecular-Weight Polyethylene
UHPC	Ultra-High-Performance Concrete
UN	United Nations
VESLMC	Very-Early Strength Latex-Modified Concrete
WPU	Waterborne Polyurethane
XRD	X-ray Diffraction
XRF	X-ray Fluorescence
